# Twelve English Statesmen

**Published:** 1890-06-14

**Authors:** 


### Twelve English Statesmen.

WILLIAM I., HENRY II., AND HENRY VII.

Messrs. Macmillan deserve the warm thanks of the reading public for planning out this excellent series* of biographies, and securing writers who, as is well known, have made the times with which they are respectively called upon to deal specially their own, and are thus enabled to present us with gems of concise and picturesque writing. To learn or re-learn our history from these brilliantly written little books is to follow out Carlyle's method more than some opponents of that worshipper of great men might perhaps approve of; but facts are stubborn things, and whether we like it or not, every candid student of history must admit that in many critical and momentous periods of her existence, England was saved, purified, and elevated through the instrumentality of one great man—a man who might even be of alien blood. Indeed, when we reflect upon the history of England, and the large part which foreigners have played in it, the question cannot but suggest itself—Would England have ever attained to her present greatness, without a large admixture of foreign blood—without what was, perhaps, even more valuable, the leadership of foreigners? May it not have been that while the Anglo-Saxon race formed an excellent basis, both in physique and in morale, something else had to be built on to it, before the English nation could fill the place to which, after the Norman conquest, it was not long in attaining?

It is evident that Mr. Freeman, who tells the story of the first of these twelve statesmen, William I., looks with no dissatisfaction on the fate which threw the English land and nation into the arms of the Norman Conqueror. We have no evidence to show that the unfortunate Harold was anything of a statesman; under him England would probably have remained as she was—divided, to all practical intents and purposes, into large and almost independent earldoms; and although she might have been spared the enmity with France into which, as Mr. Freeman thinks, her connection with Normandy led her, it would have been long before she made herself felt as a European power.

No one can pretend that William governed with any regard to the feelings and wishes of the conquered nation, except when these coincided with his own. But regarding him in the light of an invader and conqueror, and comparing what he actually did do with what he might have done, Mr. Freeman clearly thinks that we may well be grateful for our mercies. Doubtless, William was looking primarily to his own interests in taking, as he did, the utmost pains to keep the law in the letter, if not in the spirit, and, when he felt that change was necessary, to set up new enactments alongside of the old ones, rather than repeal or destroy them. But the grand result of this policy was that the conquered nation, instead of breaking unduly with its past, preserved its continuity; that the Normans gradually became English, instead of the English becoming Norman. Again, it is largely owing to the Norman invader that England gradually become a united whole. As Mr. Freeman justly observes—"William, with whatsoever motive, was, in fact, fighting for the unity of England; and it was in the long run better for Exeter, or any other part of England, to share, even in conquest, the fate of the whole land."

But it was not merely by subduing those who resisted him that William accomplished his great object. It was at the Assembly of Salisbury, held in 1086, and following after the Great Survey whose work has been preserved for all time in "Domesday Book," that the Conqueror "took the step which made England for ever one." So far from introducing the feudal system, as many do ignorantly declare, William in effect struck it a mortal blow, by declaring that duty to the king was to over-ride duty to any and every inferior lord, and calling upon every landowner, small or great, English or Norman, to swear fealty to him, and to consider himself henceforth the "man" of the king. To quote Mr. Freeman once more—"On that day England became for ever a kingdom, one and indivisible, which, since that day, no man has dreamed of parting asunder."

Henry II., whose striking personality is vividly brought before us by Mrs. J. R. Green, was, without doubt, another great statesman. When we reflect that he was not only King of England, but Duke of Normandy and Aquitaine, Count of Poitou, Anjou, Maine, and Touraine, and Suzerain Lord of Brittany, and that no common ties of custom or interest bound any of his dominions together, we are simply amazed at the indomitable energy of the man who could keep control over this heterogeneous mass of possessions, and

* "Twelve English Statesmen Series." (London: Macmillan and Co.)

yet find time for a thorough investigation of the internal affairs of his island kingdom. Moreover, the times on which he had fallen were exceptionally difficult. It was, in fact, a time of transition—a time which saw, as Mrs. Green well expresses it, "the struggle between the iron organisation of mediæval feudalism, and those nascent forces of modern civilisation which were fated in the end to shatter and supersede it." All around were signs of change and of activity—monasteries springing up all over the country, and taking the cultivation of the land into their own care; towns growing fast, and fighting hard for their liberties; foreign influences moulding the thought of the nation; a new "middle-class" springing into existence; and finally, both Church and people rapidly growing in power, and in capacity for organised resistance to the authority of the king. But if anyone could cope with the difficulties of the time, Henry was the man. He journeyed throughout the length and breadth of the land at such headlong speed, that his secretary is reduced to praying that the Almighty would "turn and convert the heart of the king from this pestilent habit," looking in at the shire courts, paying flying visits to castle and hut, drawing up charters, and thoroughly acquainting himself with all the affairs of his kingdom. Then came the work of reform. The Exchequer Court and the Curia Regis were re-organised; a new coinage was issued; the adoption of scutage gave a standingarmy to the king, and crippled the power of the barons; and the famous Assize of Clarendon, laying down the principles on which the administration of justice was to be carried out, was issued and promptly put into force. True, Henry's judges were regarded by the people as tax-gatherers rather than administrators of justice; true, Henry had a keen eye to profit, and amassed large hoards under the new system. But whether he was actuated by self-interest or regard for his people—whether he realised the full importance of his action, or whether he did not, certain it is that under him English law was administered for the first time in an orderly, methodical, and comparatively incorruptible manner.

If Henry II. was looked upon by many of his subjects as before all things a tax-gatherer, Henry VII. incurred still more odium, by his various expedients for filling his coffers. That he really was of an avaricious nature is probable; but we must be careful to remember that to a statesman-king, like this first of the Tudors, who had no notion of reigning without ruling, some command of money was essential. And although the extortions which he practised on various private persons were morally unjust as well as cruel, he may be credited with having had a better object in view than mere personal aggrandisement, viz., the prevention of that severe taxation upon the people at large to which he must otherwise have resorted.

Much of Mr. Gairdner's history of this king is taken up with foreign wars and negotiations, in which Henry certainly proved himself a great statesman, for not only did he teach his hot-headed subjects the useful lesson that if they chose to engage in war they must pay for it, but he laid the foundations, to say the least of it, of the strong and influential position which England was shortly to occupy. An unpleasant feature in his foreign policy is his ceaseless activity in devising and promoting marriages quite unsuitable from every point of view but that of political expediency. No doubt this was the fashion of the time; but surely a king who, as Mr. Gairdner reminds us, could actually propose, on the death of his wife, to marry the youthful widow of his eldest son, must have shocked the feelings of many, even in those easy-going days.

As to home policy, Henry devoted much time and care to the passing of measures to ensure the better administration of justice, and to encourage trade and commerce. True, he meddled with certain things which had better have been left alone, forbidding gold to go out of the country, regulating, or trying to regulate, the price of wool, and endeavouring to arrest the growth of sheep-farming (to which were attributed all the distresses of the time), by ordering that existing houses of husbandry must be kept up. But although this "statute of singular policy," as Bacon terms it, did not check the evils at which it was aimed, its object was, no doubt, at any rate in part, the benefit of the people; and if Henry did not fully understand political economy, that is no more than may be said of many another statesman of a later date.

